# Supplementary material for: Altered neural processing in middle frontal gyrus and cerebellum during temporal recalibration of action-outcome predictions in schizophrenia spectrum disorders
Source: Schizophrenia (Heidelb). 2026 Jan 10;12(1):10. doi: 10.1038/s41537-025-00721-y (PMC12858854; doi:10.1038/s41537-025-00721-y)
Supplement: Supplementary file 1 — Supplementary material for the research article: Altered Neural Processing in Middle Frontal Gyrus and Cerebellum During Temporal Recalibration of Action-Outcome Predictions in Schizophrenia Spectrum [file 41537_2025_721_MOESM1_ESM.docx]

**Supplementary material** for the research article:

Altered Neural Processing in Middle Frontal Gyrus and Cerebellum During Temporal Recalibration of Action-Outcome Predictions in Schizophrenia Spectrum Disorders

Christina V. Schmitter & Benjamin Straube

Department of Psychiatry and Psychotherapy, University of Marburg, Rudolf-Bultmann Strasse 8, 35039 Marburg, Germany

**S1. Details on sample characteristics**

Out of the total sample of 22 SSD participants and 19 HC, 14 SSD participants and 18 HC completed the experiment during fMRI data acquisition. The remaining participants either declined to be scanned or were unable to undergo MRI due to metallic implants. The SSD fMRI sample contained 8 participants with schizophrenia (F20) and 6 participants with schizoaffective disorder (F25). Detailed sample characteristics of the fMRI-subsamples are displayed in **Supplementary Table 1**. Furthermore, sample characteristics subdivided for the three diagnoses of the schizophrenia spectrum, namely schizophrenia (F20), schizoaffective disorder (F25), and acute polymorph psychotic disorder (F23) are provided in **Supplementary Table 2**.

**Supplementary Table 1. fMRI-Sample characteristics.**

|  | **SSD**  (N = 14) | **HC**  (N = 18) | **Group comparisons** | | |
| --- | --- | --- | --- | --- | --- |
|  |  |  | ***t*-value** | ***p*-value** | ***d*** |
| ***Demographics*** |  |  |  |  |  |
| Sex |  |  |  |  |  |
| Male | 9 (64.29%) | 8 (44.44%) |  |  |  |
| Female | 5 (35.71%) | 10 (55.56%) |  |  |  |
| Age (in years) | 36.93 ± 11.91 | 37.88 ± 10.58 | .782 | .448 | .209 |
| Education |  |  |  |  |  |
| Lower secondary | 2 (14.29%) | 1 (5.56%) |  |  |  |
| Upper secondary | 3 (21.43%) | 4 (22.22%) |  |  |  |
| Tertiary | 9 (64.29%) | 13 (72.22%) |  |  |  |
|  |  |  |  |  |  |
| ***Handedness*** |  |  |  |  |  |
| **Laterality quotient (EHI)** | **48.57 ± 60.34** | **85.00 ± 33.04** | **2.175** | **.049** | **.581** |
|  |  |  |  |  |  |
| ***Clinical measures*** |  |  |  |  |  |
| SAPS total score | 16.43 ± 13.25 |  |  |  |  |
| SANS total score | 15.21 ± 12.28 |  |  |  |  |
| BDI score | 0.55 ± 0.55 |  |  |  |  |
| GAF score | 60.07 ± 16.44 |  |  |  |  |
| SOFAS score | 75.86 ± 15.29 |  |  |  |  |
|  |  |  |  |  |  |
| ***Neuropsychological control measures*** | | | | | |
| Attention |  |  |  |  |  |
| d2 score | 159.64 ± 18.61 | 174.69 ± 48.03 | .528 | .607 | .147 |
| Executive functions |  |  |  |  |  |
| TMT-A (sec.) | 25.77 ± 7.94 | 24.04 ± 9.81 | -.235 | .818 | -.063 |
| TMT-B (sec.) | 73.89 ± 25.25 | 56.51 ± 23.32 | -1.587 | .137 | -.424 |
| Short term memory |  |  |  |  |  |
| WAIS: FS score | 7.36 ± 1.50 | 7.33 ± 1.53 | -.434 | .671 | -.116 |
| WAIS: BS score | 5.85 ± 0.95 | 6.72 ± 1.27 | 1.710 | .111 | .457 |
|  |  |  |  |  |  |
| ***Antipsychotic medication*** |  |  |  |  |  |
| None | 3 | 18 |  |  |  |
| First generation | 0 | 0 |  |  |  |
| Second generation | 11 | 0 |  |  |  |

d2: d2 test of attention,^1^ TMT: Trial Making Test,^2^ WAIS: Wechsler Adult Intelligence Scale,^3^ FS: Forward span, BS: Backward span, SAPS: Scale for the Assessment of Positive Symptoms,^4^ SANS: Scale for the Assessment of Negative Symptoms,^5^ BDI: Beck Depression Inventory,^6^ GAF: Global Assessment of Functioning,^7^ SOFAS: Social and Occupational Functioning Assessment Scale.^8^ For continuous variables the mean +/- standard deviation is displayed. Significant differences between groups are highlighted in bold.

**Supplementary Table 2. Sample characteristics of all SSD diagnoses.**

|  | **F20**  (N = 13) | **F25**  (N = 7) | **F23**  (N = 2) |
| --- | --- | --- | --- |
| ***Demographics*** | | | |
| Sex |  |  |  |
| Male | 9 (69.23%) | 2 (28.57%) | 0 |
| Female | 4 (30.77%) | 5 (71.43%) | 2 (100%) |
| Age (in years) | 36.69 ± 8.34 | 36.43 ± 15.59 | 25.0 ± 2.83 |
| Education |  |  |  |
| Lower secondary | 1 (7.69%) | 1 (14.29%) | 0 |
| Upper secondary | 5 (38.46%) | 0 | 0 |
| Tertiary | 7 (53.85%) | 6 (85.71%) | 2 (100%) |
|  |  |  |  |
| ***Handedness*** | | | |
| Laterality quotient (EHI) | 55.38 ± 43.96 | 62.86 ± 68.60 | 85.0 ± 5.0 |
|  |  |  |  |
| ***Clinical measures*** |  |  |  |
| SAPS total score | 20.15 ± 13.62 | 9.43 ± 5.65 | 11.0 ± 2.83 |
| SANS total score | 15.0 ± 13.78 | 10.71 ± 8.20 | 3.50 ± 4.95 |
| BDI score | 0.64 ± 0.49 | 0.35 ± 0.41 | 0.26 ± 0.03 |
| GAF score | 57.85 ± 17.77 | 66.57 ± 7.57 | 85.0 ± 14.14 |
| SOFAS score | 78.38 ± 13.89 | 76.86 ± 15.46 | 92.5 ± 3.53 |
|  |  |  |  |
| ***Neuropsychological control measures*** | | | |
| Attention |  |  |  |
| d2 score | 169.61 ± 46.29 | 154.57 ± 21.48 | 178.50 ± 41.72 |
| Executive functions |  |  |  |
| TMT-A (sec.) | 23.55 ± 6.59 | 29.0 ± 7.95 | 20.44 ± 7.64 |
| TMT-B (sec.) | 71.68 ± 29.95 | 72.29 ± 20.74 | 71.28 ± 21.64 |
| Short term memory |  |  |  |
| WAIS: FS score | 7.38 ± 1.50 | 8.43 ± 1.40 | 9.5 ± 2.12 |
| WAIS: BS score | 5.92 ± 1.60 | 6.28 ± 0.75 | 6.0 ± 0.0 |
|  |  |  |  |
| ***Antipsychotic medication*** | | | |
| None | 2 | 1 | 0 |
| First generation | 2^b^ | 0 | 0 |
| Second generation | 11^b^ | 6 | 2 |

^b^ Two SSD participants were treated with both first- and second-generation antipsychotics. For continuous variables the mean +/- standard deviation is displayed. Due to the small sample size, we did not test for significant differences between the diagnostic subgroups. Please also note that exploratory re-analyses excluding the two F23 patients resulted in no differences from the reported results of the main manuscript.

**S2. Training Procedure**

To ensure correct execution of button presses and familiarize participants with the task, all participants underwent a training session before performing the fMRI experiment. The training procedure for this experimental task has been described in detail previously.^9^ Participants practiced allowing their finger to be moved passively by the button device without applying counter-pressure and learned to execute button presses with precise timing – approx. every 800ms during adaptation phases and lasting around 500ms in both adaptation and test phases. The 500ms duration ensured that stimuli were always presented before the button‘s upward movement, preventing potential interference with delay detection. Each adaptation phase ended automatically after nine button presses per segment. If participants pressed too quickly (faster than 8000ms in total), the inter-phase jitter (if in the first segment) or the instruction display before the test phase (if in the second segment) was extended accordingly. To further familiarize themselves with the task, participants practiced test phases for each condition – once without a delay and once with the maximum delay (417ms). During this training, they received feedback on whether a delay was present. They were instructed to respond as accurately as possible, without time pressure. Finally, they completed a 10-minute training session of the full experiment.

**S3. Detailed summary of behavioral results.**

To test for recalibration effects after exposure to the auditory adaptation delay on auditory perception (unimodal trials) and its transfer to visual perception (cross-modal trials), detection thresholds, slopes, and widths of the psychometric functions were analyzed using mixed ANOVAs for the respective trials. Furthermore, Bayes factors (BF_incl_) were calculated for all effects. The results of these analyses are summarized in **Supplementary Tables 3 and 4.**

**Supplementary Table 3. Results of the ANOVA on detection thresholds, slopes, and widths of the psychometric functions in unimodal trials (auditory test modality).**

| **Effect** | | **df** | **F** | **p** | **η²_p_** | **BF_incl_** |  |
| --- | --- | --- | --- | --- | --- | --- | --- |
| **Detection thresholds** | |  |  |  |  |  |  |
|  | Group | 1 | .415 | .523 | .011 | .648 |  |
|  | *Residuals* | 39 |  |  |  |  |  |
|  | Movement type | 1 | 2.807 | .102 | .067 | .707 |  |
|  | **Movement type ✻ Group** | **1** | **4.988** | **.031** | **.113** | **1.214** |  |
|  | *Residuals* | 39 |  |  |  |  |  |
|  | **Adaptation delay** | **1** | **15.191** | **< .001** | **.280** | **18.351** |  |
|  | Adaptation delay ✻ Group | 1 | < .001 | .994 | < .001 | .363 |  |
|  | *Residuals* | 39 |  |  |  |  |  |
|  | Movement type ✻ Adaptation delay | 1 | .768 | .386 | .019 | .439 |  |
|  | Movement type ✻ Adaptation delay ✻ Group | 1 | 2.988 | .092 | .071 | .268 |  |
|  | *Residuals* | 39 |  |  |  |  |  |
| **Slopes** | |  |  |  |  |  |  |
|  | Group | 1 | 4.056 | .051 | .094 | .638 |  |
|  | *Residuals* | 39 |  |  |  |  |  |
|  | Movement type | 1 | 3.461 | .070 | .082 | .474 |  |
|  | Movement type ✻ Group | 1 | < .001 | .977 | < .001 | .214 |  |
|  | *Residuals* | 39 |  |  |  |  |  |
|  | Adaptation delay | 1 | 1.018 | .319 | .025 | .143 |  |
|  | Adaptation delay ✻ Group | 1 | .073 | .789 | .002 | .096 |  |
|  | *Residuals* | 39 |  |  |  |  |  |
|  | Movement type ✻ Adaptation delay | 1 | .528 | .472 | .013 | .113 |  |
|  | Movement type ✻ Adaptation delay ✻ Group | 1 | .154 | .697 | .004 | .009 |  |
|  | *Residuals* | 39 |  |  |  |  |  |
| **Widths** | |  |  |  |  |  |  |
|  | Group | 1 | 2.552 | .118 | .061 | .391 |  |
|  | *Residuals* | 39 |  |  |  |  |  |
|  | Movement type | 1 | 4.001 | .052 | .093 | .545 |  |
|  | Movement type ✻ Group | 1 | .039 | .844 | .001 | .172 |  |
|  | *Residuals* | 39 |  |  |  |  |  |
|  | Adaptation delay | 1 | .332 | .568 | .008 | .091 |  |
|  | Adaptation delay ✻ Group | 1 | .089 | .767 | .002 | .058 |  |
|  | *Residuals* | 39 |  |  |  |  |  |
|  | Movement type ✻ Adaptation delay | 1 | .035 | .853 | < .001 | .058 |  |
|  | Movement type ✻ Adaptation delay ✻ Group | 1 | .468 | .498 | .012 | .005 |  |
|  | *Residuals* | 39 |  |  |  |  |  |

 N_SSD_ = 22, N_HC_ = 19. Significant effects (*p* < .05) are highlighted in bold.

**Supplementary Table 4. Results of the ANOVA on detection thresholds, slopes, and widths of the psychometric functions in cross-modal trials (visual test modality).**

| **Effect** | | **df** | **F** | **p** | **η²_p_** | **BF_incl_** |  |
| --- | --- | --- | --- | --- | --- | --- | --- |
| **Detection thresholds** | |  |  |  |  |  |  |
|  | Group | 1 | < .001 | .983 | < .001 | .673 |  |
|  | *Residuals* | 39 |  |  |  |  |  |
|  | **Movement type** | **1** | **4.296** | **.045** | **.099** | **19.301** |  |
|  | Movement type ✻ Group | 1 | 1.207 | .279 | .030 | .846 |  |
|  | *Residuals* | 39 |  |  |  |  |  |
|  | **Adaptation delay** | **1** | **8.302** | **.006** | **.176** | **30.611** |  |
|  | Adaptation delay ✻ Group | 1 | .082 | .776 | .002 | .503 |  |
|  | *Residuals* | 39 |  |  |  |  |  |
|  | **Movement type ✻ Adaptation delay** | **1** | **10.532** | **.002** | **.213** | **31.895** |  |
|  | Movement type ✻ Adaptation delay ✻ Group | 1 | .843 | .364 | .021 | .485 |  |
|  | *Residuals* | 39 |  |  |  |  |  |
| **Slopes** | |  |  |  |  |  |  |
|  | Group | 1 | .156 | .695 | .004 | .208 |  |
|  | *Residuals* | 39 |  |  |  |  |  |
|  | **Movement type** | **1** | **8.573** | **.006** | **.180** | **2.286** |  |
|  | Movement type ✻ Group | 1 | 3.096 | .086 | .074 | .445 |  |
|  | *Residuals* | 39 |  |  |  |  |  |
|  | Adaptation delay | 1 | .136 | .714 | .003 | .128 |  |
|  | Adaptation delay ✻ Group | 1 | .343 | .561 | .009 | .062 |  |
|  | *Residuals* | 39 |  |  |  |  |  |
|  | Movement type ✻ Adaptation delay | 1 | .520 | .475 | .013 | .120 |  |
|  | Movement type ✻ Adaptation delay ✻ Group | 1 | .138 | .712 | .004 | .014 |  |
|  | *Residuals* | 39 |  |  |  |  |  |
| **Widths** | |  |  |  |  |  |  |
|  | Group | 1 | .007 | .935 | < .001 | .232 |  |
|  | *Residuals* | 39 |  |  |  |  |  |
|  | **Movement type** | **1** | **12.174** | **.001** | **.238** | **9.954** |  |
|  | Movement type ✻ Group | 1 | 2.031 | .162 | .050 | .443 |  |
|  | *Residuals* | 39 |  |  |  |  |  |
|  | Adaptation delay | 1 | .252 | .619 | .006 | .145 |  |
|  | Adaptation delay ✻ Group | 1 | .800 | .377 | .020 | .085 |  |
|  | *Residuals* | 39 |  |  |  |  |  |
|  | Movement type ✻ Adaptation delay | 1 | .533 | .470 | .013 | .140 |  |
|  | Movement type ✻ Adaptation delay ✻ Group | 1 | .050 | .824 | .001 | .021 |  |
|  | *Residuals* | 39 |  |  |  |  |  |

N_SSD_ = 22, N_HC_ = 19. Significant effects (*p* < .05) are highlighted in bold.

**S4. Main effect of Movement Type (Active > Passive)**

To determine whether motor-related processes were specific to active movement conditions, we compared brain activation between active and passive conditions (Active > Passive) in test phases. As expected, active conditions elicited stronger activation in motor-related regions, particularly in the left precentral gyrus and the cerebellum (see **Supplementary Fig. 1** and **Supplementary Table 5**). This suggests that motor-related predictive processes that were of primary interest in our study should be specific to active conditions.


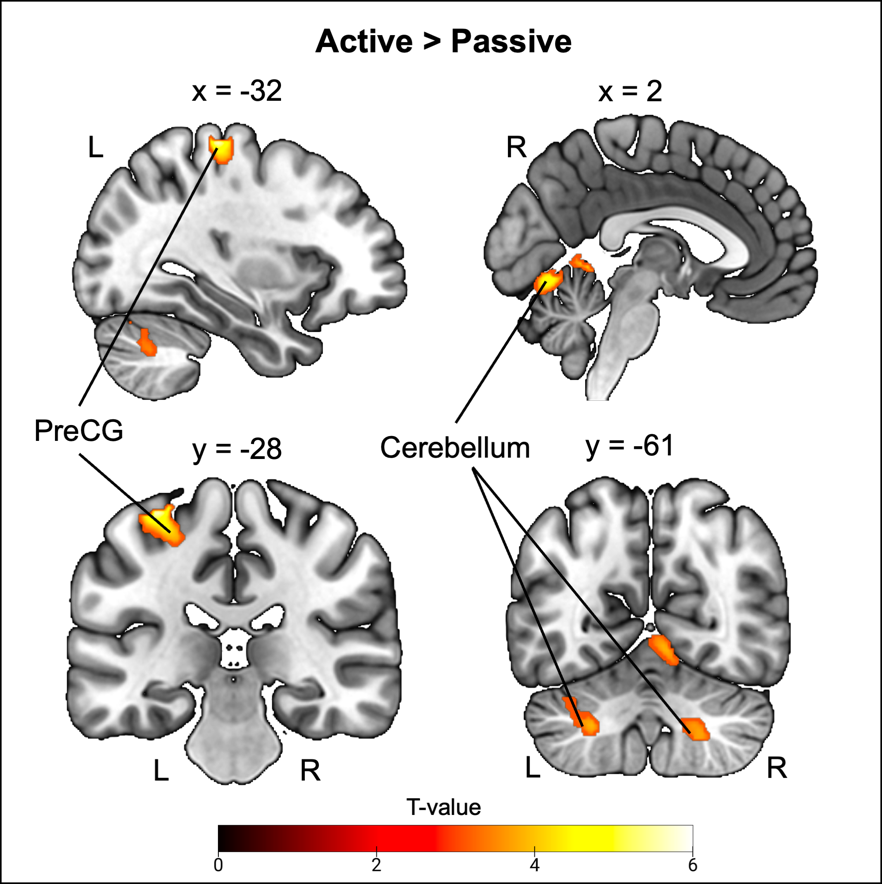


**Supplementary Fig. 1. Group results for the main effect of movement type (Active > Passive).** In test phases, active movements were associated with increased activity in left precentral gyrus and bilateral cerebellum. PreCG = precentral gyrus, L = left, R = right.

**Supplementary Table 5. Group results for the main effect of movement type (Active > Passive).**

| **Cluster peak** | **Local**  **peaks** | **Hem.** | **Coordinates** | | | **T-value** | **no.**  **voxels** |
| --- | --- | --- | --- | --- | --- | --- | --- |
|  |  |  | **x** | **y** | **z** |  |  |
| **PreCG** |  | **L** | **-32** | **-28** | **66** | **5.92** | **234** |
| **Vermis VI** |  | **.** | **2** | **-70** | **-10** | **5.73** | **518** |
|  | Vermis IV/V | . | 0 | -50 | -2 | 4.53 |  |
| **CER VIII** |  | **R** | **22** | **-58** | **-44** | **4.19** | **137** |
| **CER IV/V** |  | **R** | **14** | **-46** | **-12** | **4.05** | **128** |
|  | CER VI | R | 30 | -46 | -24 | 3.87 |  |
| **CER VI** |  | **L** | **-26** | **-74** | **-22** | **4.01** | **266** |
|  | CER Crus I | L | -26 | -62 | -40 | 3.99 |  |
|  | CER Crus I | L | -36 | -64 | -30 | 3.44 |  |

N_SSD_ = 14, N_HC_ = 18. Coordinates are listed in MNI space. Significance level: p < .001 uncorrected with a minimum cluster extent of 99 voxels (p < .05 Monte Carlo cluster level corrected). CER = cerebellum, PreCG = precentral gyrus, L = left, R = right.

**S5. Details on correlation analyses between recalibration effects and symptom severity**

Exploratory correlation analyses were performed to investigate the association between symptom severity, assessed via the SAPS total score and subscores for hallucinations and delusions, and both behavioral and neural TREs. A summary of all correlation results is provided in **Supplementary Table 6**. Scatter plots for significant correlations are provided in **Supplementary Fig. 2**.

**Supplementary Table 6. Correlations between behavioral and neural TREs and symptom severity**

| **Behavioral TRE** | | | | | | | | | | | | | | | | |
| --- | --- | --- | --- | --- | --- | --- | --- | --- | --- | --- | --- | --- | --- | --- | --- | --- |
| **TRE per Condition** | **r-value** | | | **p-value** | | | | **90% Bootstrapped CI** | | | | | | | | |
|  |  | | |  | | | | **low** | | | | **high** | | | | |
|  | ***Tot*** | ***Hal*** | ***Del*** | ***Tot*** | | ***Hal*** | ***Del*** | ***Tot*** | | ***Hal*** | ***Del*** | ***Tot*** | | ***Hal*** | | ***Del*** |
| Active/Auditory | -.072 | -.100 | -.096 | .749 | | .658 | .670 | -.586 | | -.459 | -.525 | .434 | | .316 | | .293 |
| Passive/Auditory | .130 | .143 | .137 | .563 | | .526 | .543 | -.265 | | -.159 | -.264 | .440 | | .376 | | .481 |
| Active/Visual | .197 | -.027 | .375 | .380 | | .904 | .086 | -.074 | | -.311 | .088 | .485 | | .254 | | .612 |
| Passive/Visual | -.094 | -.009 | -.064 | .678 | | .969 | .777 | -.380 | | -.309 | -.355 | .202 | | .267 | | .186 |
| **Neural TRE** | | | | | | | | | | | | | | | | |
| **TRE per Contrast** | **r-value** | | | **p-value** | | | | **90% Bootstrapped CI** | | | | | | | | |
|  |  | | |  | | | | **low** | | | | **high** | | | | |
|  | ***Tot*** | ***Hal*** | ***Del*** | ***Tot*** | | ***Hal*** | ***Del*** | ***Tot*** | | ***Hal*** | ***Del*** | ***Tot*** | | ***Hal*** | | ***Del*** |
| ***Auditory: 0ms > 200ms*** | | | | | | | | | | | | | | | | |
| Cluster 1 (PostCG) | .027 | -.083 | -.099 | .927 | | .777 | .735 | -.636 | | -.667 | -.692 | .658 | | .404 | | .588 |
| Cluster 2 (SFG) | -.108 | -.149 | -.144 | .712 | | .612 | .623 | -.534 | | -.494 | -.557 | .372 | | .281 | | .293 |
| Cluster 3 (SFG) | .022 | -.176 | -.041 | .942 | | .547 | .890 | -.448 | | -.663 | -.536 | .511 | | .362 | | .553 |
| ***Auditory: Group x Adaptation delay x Movement type*** | | | | | | | | | | | | | | | | |
| Cluster 1 (MFG) | -.046 | .083 | .009 | .875 | | .777 | .976 | -.478 | | -.294 | -.458 | .579 | | .733 | | .443 |
| ***Visual: Adaptation delay x Movement type*** | | | | | | | | | | | | | | | | |
| Cluster 1 (TPOsup) | .337 | .270 | -.082 | .239 | | .351 | .780 | -.039 | | -.104 | -.560 | .650 | | .639 | | .362 |
| **Cluster 2 (PUT)** | **.690** | **.715** | .297 | **.006** | | **.004** | .303 | **.084** | | **.386** | -.657 | **.870** | | **.865** | | .699 |
| **Cluster 3 (HES)** | **.581** | **.611** | .239 | **.029** | | **.020** | .410 | **.075** | | **.214** | -.541 | **.831** | | **.818** | | .657 |
| Cluster 4 (CAL) | .313 | .445 | -.040 | .276 | | .111 | .893 | -.192 | | .074 | -.728 | .699 | | .757 | | .420 |
| Cluster 5 (MTG) | .222 | .343 | -.186 | .446 | | .230 | .525 | -.186 | | -.017 | -.682 | .524 | | .630 | | .294 |
| Cluster 6 (PFC) | .167 | .179 | -.229 | .569 | | .539 | .430 | -.314 | | -.456 | -.587 | .507 | | .586 | | .140 |
| Cluster 7 (INS) | .423 | .419 | .059 | .132 | | .136 | .841 | .096 | | .002 | -.458 | .708 | | .713 | | .560 |
| ***Visual: Group x Adaptation delay x Movement type*** | | | | | | | | | | | | | | | | |
| Cluster 1 (CER VI) | .005 | .303 | -.079 | | .986 | .293 | .789 | | -.427 | -.253 | -.485 | | .581 | .771 | .337 | |

Behavioral data: N_SSD_ = 22, fMRI data: N_SSD_ = 14. For each condition or cluster, three Pearson’s r-values, p-values, and 90% bootstrapped confidence intervals are reported, corresponding to the correlation of the respective TRE with the SAPS total score (Tot), SAPS score 1 for hallucinations (Hal), and SAPS score 2 for delusions (Del). Significant correlations are highlighted in bold. PostCG = postcentral gyrus, SFG = superior frontal gyrus, MFG = middle frontal gyrus, TPOsup = Temporal pole: superior temporal gyrus, PUT = putamen, HES = Heschl’s gyrus, CAL = Calcarine, MTG = middle temporal gyrus, PFCventmed = Superior frontal gyrus: medial orbital, INS = insula, CER = cerebellum.


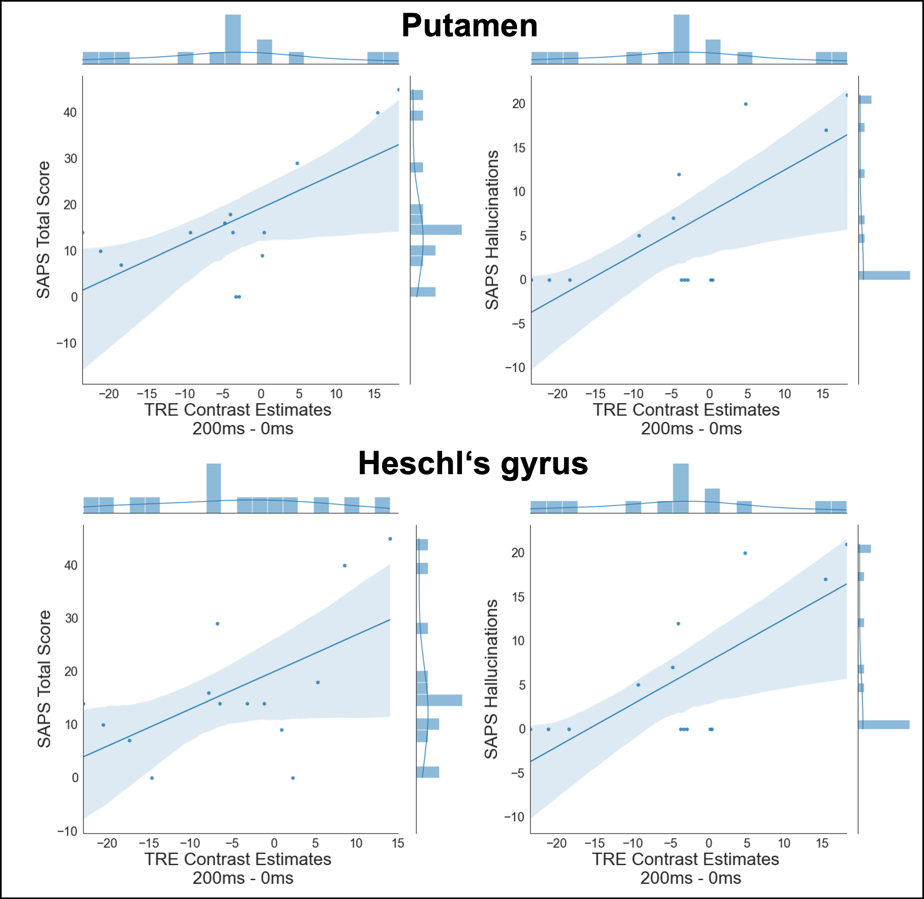


**Supplementary Fig. 2. Scatter plots for significant correlations of TREs and symptom severity.** SSD participants with a higher SAPS total score and a higher hallucinations subscore were the ones that deviated most strongly from the overall activation pattern in active conditions (i.e., a negative neural TRE) observed across both groups in putamen and Heschl’s gyrus (visual test modality; contrast: Adaptation delay x Movement type).

**S6. Exploratory analysis of delay-dependent activations during adaptation phases**

We used a parametric analysis to identify brain regions which exhibited a linear change in activation throughout the adaptation phases. This analysis relied on the same preprocessed data that were used in the main manuscript and on the same definition of regressors in the GLM for the experimental conditions and events of no interest. As described in the main manuscript, for the adaptation phases, regressors were defined based on eight experimental conditions comprising the factors *adaptation delay* (0ms, 200ms), *movement type* (active, passive), and *adaptation phase* (early, late). To parametrically modulate these regressors, the number of button presses performed during early adaptation phases (i.e., before the presentation of the fixation cross) and during late phases (i.e., after the fixation cross) were used as parametric regressor. For the single-participant GLMs, T-maps were generated by contrasting each of the eight parametric regressors against the implicit baseline. For group-level analyses, the resulting contrast estimates from each participant were entered into a flexible factorial design. As in the analysis reported in the main manuscript, we were interested in main and interaction contrasts involving the *adaptation delay* factor.

We first examined differences in parametric brain activation changes across both groups. Here, the significant interaction contrast composed of the factors *adaptation delay* and *movement type* revealed that activation in right supplementary motor area, right caudate, and in left cerebellum decreased linearly throughout adaptation when participants were exposed to the 200ms delay (see **Supplementary Fig. 3** and **Supplementary Table 7**). This effect was comparatively stronger in active movement conditions than in passive ones. The SMA is thought to be involved in generating the efference copy of motor commands, which is then projected to the cerebellum. The cerebellum uses this efference copy to perform forward model operations by predicting the sensory outcomes of the motor commands. If necessary, the prediction error is then sent back to the SMA to adjust the motor commands for future actions.^10,11^ It has already been shown before that the connectivity between the cerebellum and the SMA temporarily increases when we are exposed to an additional delay between action and action-outcome.^12^ Therefore, it is likely that at the beginning of the adaptation phases, a stronger prediction error signal is present in the cerebellum and SMA, which then gradually diminishes as the forward model integrates the constant delay into its predictions.

To investigate whether parametric brain activation changes during adaptation differed between participants with SSD and HC, we examined contrasts involving the factors *group* and *adaptation delay*. A significant four-way interaction of *group*, *adaptation delay*, *movement type* and *adaptation phase* emerged, with a cluster spanning left precuneus and right posterior cingulate cortex. In these regions, HC showed a linear increase of activation in late adaptation phases while exposed to the 200ms delay, with a stronger effect in active than in passive conditions. For SSD, a similar activation increase in these regions could be observed during early adaptation phases. Both regions are part of a network of cortical midline structures which are involved in processing self-referential information and have been associated with the experience of agency.^13,14^ It appears that these regions show stronger activation in HC when they perceive synchrony between action and outcome or when the outcome is attributed to their own action, which is likely to occur in later phases of adaptation.^15^ In SSD, functional alterations in the precuneus during self-referential processing are well-documented^16^ and may, in this case, lead to deviations in the process of attributing the delayed outcome to the own action compared to HC. However, the precise nature of these activity deviations and their potential consequences remain open based on the present data.


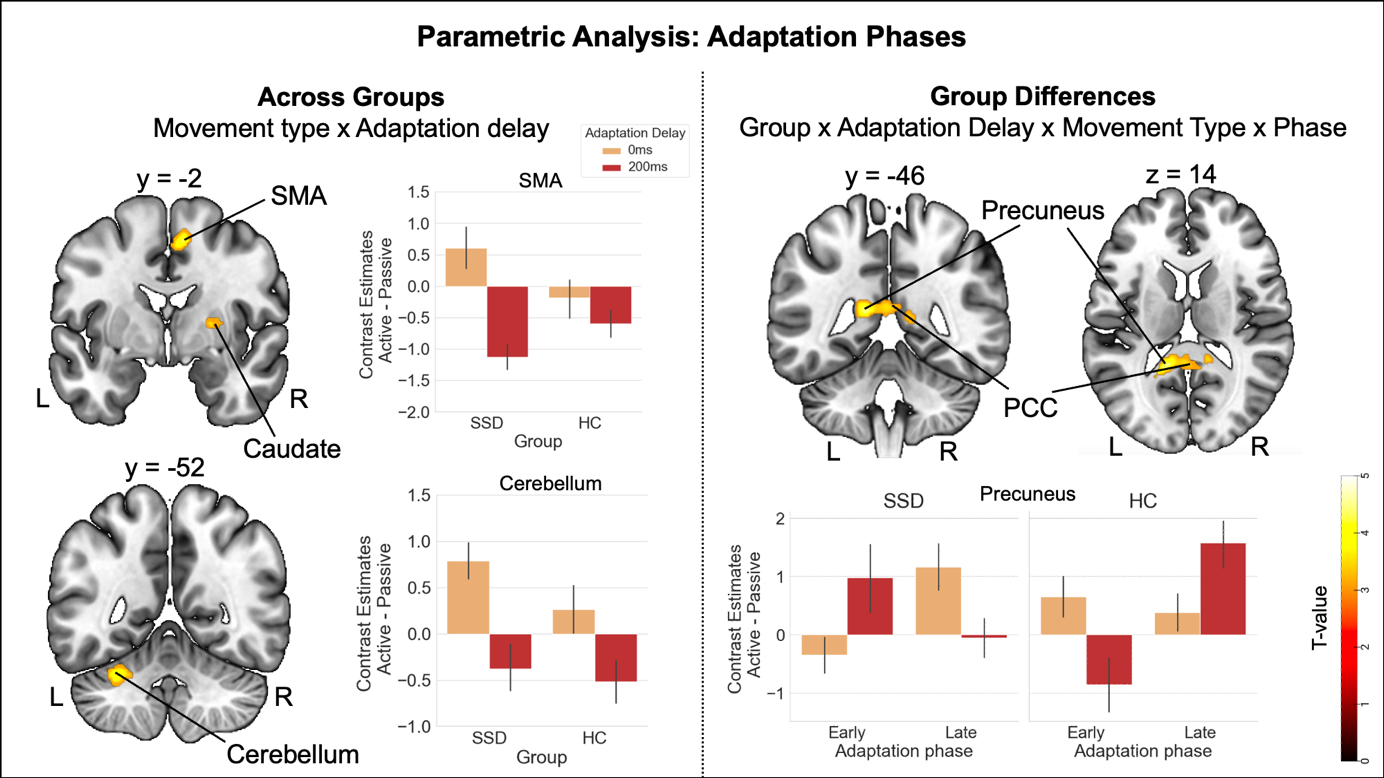


**Supplementary Fig. 3. Group results** **for the parametric analysis of adaptation phases. Left panel:** Parametric analyses across groups showed a decrease in activation over the course of adaptation to the 200ms delay in right supplementary motor area, right caudate, and left cerebellum. This effect was comparatively stronger in active movement conditions than in passive ones. **Right panel:** Group differences emerged in left precuneus and right posterior cingulate cortex where HC show an activation increase in late adaptation phases while exposed to the 200ms delay, with a stronger effect in active than in passive conditions. For SSD, this activation increase occurred during early adaptation phases. For visualization, contrast estimates (eigenvariates extracted with the VOI function of SPM) are displayed as the difference between active and passive conditions, emphasizing the extent to which the effect is more pronounced in active compared to passive conditions. Error bars show standard errors of the mean. SMA = supplementary motor area, PCC = posterior cingulate cortex, L = left, R = right.

**Supplementary Table 7. Group results for the parametric analysis of adaptation phases**

| **Cluster peak** | **Local**  **peaks** | **Hem.** | **Coordinates** | | | **T-value** | **no.**  **voxels** |
| --- | --- | --- | --- | --- | --- | --- | --- |
|  |  |  | **x** | **y** | **z** |  |  |
| **Across Groups: Interaction Adaptation delay x Movement type** | | | | | | | |
| **SMA** |  | **R** | **8** | **-2** | **54** | **4.42** | **103** |
| **CAU** |  | **R** | **18** | **8** | **16** | **4.22** | **132** |
|  | PUT | R | 24 | 0 | 6 | 3.83 |  |
| **CER VI** |  | **L** | **-30** | **-52** | **-28** | **4.19** | **100** |
|  | CER IV/V | L | -18 | -48 | -30 | 3.35 |  |
| **Interaction Group x Adaptation delay x Movement type x Phase** | | | | | | | |
| **PCUN** |  | **L** | **-14** | **-46** | **14** | **4.60** | **342** |
|  | PCC | R | 12 | -42 | 10 | 3.85 |  |
|  | PCC | R | 4 | -36 | 6 | 3.75 |  |

N_SSD_ = 14, N_HC_ = 18. Coordinates are listed in MNI space. Significance level: p < .001 uncorrected with a minimum cluster extent of 99 voxels (p < .05 Monte Carlo cluster level corrected). SMA = supplementary motor area, CAU = caudate, PUT = putamen, CER = cerebellum, PCUN = precuneus, PCC = posterior cingulate cortex, L = left, R = right.

**S7. Exploratory correlation analyses between chlorpromazine (CPZ) equivalents and behavioral and neural recalibration effects**

To investigate whether the observed effects were influenced by antipsychotic medication, we conducted exploratory correlations between the CPZ equivalents (mg/d) and both behavioral and neural TREs. To convert the medication doses into CPZ equivalents, we used the “chlorpromazineR” package (version 0.2.0) for R^17^ (version 4.2.2). The conversion factors of aripiprazole, quetiapine, olanzapine, clozapine, amisulpride, risperidone, paliperidone, and flupenthixol were based on the work of Gardner et al. (2010)^18^, while the conversion factors for melperone and cariprazine were taken from Leucht et al. (2016)^19^ and Leucht et al. (2020),^20^ respectively. On the behavioral level, CPZ equivalents (*Mean* = 419.294, *SD* = 435.893) were then correlated with the TREs for each experimental condition. On the neural level, CPZ equivalents (*Mean* = 426.633, *SD* = 518.425) were correlated with the TREs of the contrast estimates (200ms – 0ms) of each cluster for significant fMRI contrasts involving the *adaptation delay* factor following a similar logic as described for the correlation analyses with symptom severity reported in the main manuscript. None of the correlations reached significance (see **Supplementary Table 8** for a detailed summary of all correlation results), suggesting that the medication did not interfere with recalibration processes and performance in the task.

**Supplementary Table 8. Correlations between behavioral and neural TREs and CPZ equivalents**

| **Behavioral TRE** | | | | |
| --- | --- | --- | --- | --- |
| **TRE per Condition** | **r-value** | **p-value** | **90% Bootstrapped CI** | |
|  |  |  | **low** | **high** |
| Active/Auditory | .182 | .417 | -.084 | .406 |
| Passive/Auditory | -.074 | .744 | -.475 | .212 |
| Active/Visual | .180 | .422 | -.046 | .378 |
| Passive/Visual | .135 | .549 | -.269 | .494 |
| **Neural TRE** | | | | |
| **TRE per Contrast** | **r-value** | **p-value** | **90% Bootstrapped CI** | |
|  |  |  | **low** | **high** |
| ***Auditory: 0ms > 200ms*** | | | | |
| Cluster 1 (PostCG) | .304 | .290 | -.025 | .542 |
| Cluster 2 (SFG) | .498 | .070 | .081 | .779 |
| Cluster 3 (SFG) | .240 | .409 | -.293 | .645 |
| ***Auditory: Group x Adaptation delay x Movement type*** | | | | |
| Cluster 1 (MFG) | .257 | .376 | -.509 | .861 |
| ***Visual: Adaptation delay x Movement type*** | | | | |
| Cluster 1 (TPOsup) | .011 | .971 | -.433 | .482 |
| Cluster 2 (PUT) | -.272 | .347 | -.746 | .232 |
| Cluster 3 (HES) | -.254 | .380 | -.662 | .306 |
| Cluster 4 (CAL) | -.193 | .509 | -.771 | .423 |
| Cluster 5 (MTG) | -.075 | .798 | -.730 | .481 |
| Cluster 6 (PFC) | -.079 | .789 | -.595 | .500 |
| Cluster 7 (INS) | -.337 | .239 | -.788 | .144 |
| ***Visual: Group x Adaptation delay x Movement type*** | | | | |
| Cluster 1 (CER VI) | -.234 | .421 | -.703 | .757 |

Behavioral data: N_SSD_ = 22, fMRI data: N_SSD_ = 14. For each condition or cluster, Pearson’s r-values, p-values, and 90% bootstrapped confidence intervals are reported, corresponding to the correlation of the respective TRE with the CPZ equivalents. PostCG = postcentral gyrus, SFG = superior frontal gyrus, MFG = middle frontal gyrus, TPOsup = Temporal pole: superior temporal gyrus, PUT = putamen, HES = Heschl’s gyrus, CAL = Calcarine, MTG = middle temporal gyrus, PFCventmed = Superior frontal gyrus: medial orbital, INS = insula, CER = cerebellum.

**References**

1. Brickenkamp R. *D2 Aufmerksamkeits-Belastungs-Test.* 8th ed. Göttingen: Hogrefe; 1994.

2. Reitan RM. *Trail Making Test*. Tucson, AZ: Reitan Neuropsychology Laboratory; 1992.

3. Petermann F. *WAIS-IV. Wechsler Adult Intelligence Scale*. Frankfurt: Pearson; 2012.

4. Andreasen NC. *The Scale for the Assessment of Positive Symptoms (SAPS)*. Iowa City: The University of Iowa; 1984.

5. Andreasen NC. *The Scale for the Assessment of Negative Symptoms (SANS)*. Iowa City: The University of Iowa; 1983.

6. Beck AT, Steer RA, Brown G. *Beck Depression Inventory–II (BDI-II)*. APA PsycTests; 1996.

7. Hall RCW. Global Assessment of Functioning. *Psychosomatics*. 1995;36(3):267-275. doi:10.1016/S0033-3182(95)71666-8

8. Goldman HH, Skodol AE, Lave TR. Revising axis V for DSM-IV: a review of measures of social functioning. *Am J Psychiatry*. 1992;149(9):1148-1156. doi:10.1176/ajp.149.9.1148

9. Schmitter CV, Straube B. Facilitation of sensorimotor temporal recalibration mechanisms by cerebellar tDCS in patients with schizophrenia spectrum disorders and healthy individuals. *Sci Rep*. 2024;14(1):2627. doi:10.1038/s41598-024-53148-3

10. Haggard P, Whitford B. Supplementary motor area provides an efferent signal for sensory suppression. *Cogn Brain Res*. 2004;19(1):52-58. doi:10.1016/j.cogbrainres.2003.10.018

11. Welniarz Q, Worbe Y, Gallea C. The Forward Model: A Unifying Theory for the Role of the Cerebellum in Motor Control and Sense of Agency. *Front Syst Neurosci*. 2021;15:644059. doi:10.3389/fnsys.2021.644059

12. Kilteni K, Houborg C, Ehrsson HH. Brief Temporal Perturbations in Somatosensory Reafference Disrupt Perceptual and Neural Attenuation and Increase Supplementary Motor Area–Cerebellar Connectivity. *J Neurosci*. 2023;43(28):5251-5263. doi:10.1523/JNEUROSCI.1743-22.2023

13. Northoff G, Heinzel A, de Greck M, Bermpohl F, Dobrowolny H, Panksepp J. Self-referential processing in our brain—A meta-analysis of imaging studies on the self. *NeuroImage*. 2006;31(1):440-457. doi:10.1016/j.neuroimage.2005.12.002

14. Farrer C, Frith CD. Experiencing Oneself vs Another Person as Being the Cause of an Action: The Neural Correlates of the Experience of Agency. *NeuroImage*. 2002;15(3):596-603. doi:10.1006/nimg.2001.1009

15. Schmitter CV, Kufer K, Steinsträter O, Sommer J, Kircher T, Straube B. Neural correlates of temporal recalibration to delayed auditory feedback of active and passive movements. *Hum Brain Mapp*. Published online October 11, 2023:hbm.26508. doi:10.1002/hbm.26508

16. Larivière S, Lavigne KM, Woodward TS, Gerretsen P, Graff-Guerrero A, Menon M. Altered functional connectivity in brain networks underlying self-referential processing in delusions of reference in schizophrenia. *Psychiatry Res Neuroimaging*. 2017;263:32-43. doi:10.1016/j.pscychresns.2017.03.005

17. R Core Team. R: A language and environment for statistical computing. R Foundation for Statistical Computing, Vienna, Austria. URL https://www.R-project.org/. Published online 2021.

18. Gardner DM, Murphy AL, O’Donnell H, Pharm B, Centorrino F, Baldessarini RJ. International Consensus Study of Antipsychotic Dosing. *Am J Psychiatry*. Published online 2010.

19. Leucht S, Samara M, Heres S, Davis JM. Dose Equivalents for Antipsychotic Drugs: The DDD Method. *Schizophr Bull*. 2016;42(suppl 1):S90-S94. doi:10.1093/schbul/sbv167

20. Leucht S, Crippa A, Siafis S, Patel MX, Orsini N, Davis JM. Dose-Response Meta-Analysis of Antipsychotic Drugs for Acute Schizophrenia. *Am J Psychiatry*. 2020;177(4):342-353. doi:10.1176/appi.ajp.2019.19010034
